# Incidental screening of descending colon carcinoma by 18F-FDG PET/MR imaging in a patient with endometrial carcinoma: A case report of Lynch syndrome

**DOI:** 10.3389/fonc.2022.1115056

**Published:** 2023-01-25

**Authors:** Xiaoran Li, Xian Zhang, Bixiao Cui, Jie Ma, Shijun Wang, Jie Lu

**Affiliations:** ^1^ Department of Radiology and Nuclear Medicine, Xuanwu Hospital, Capital Medical University, Beijing, China; ^2^ Beijing Key Laboratory of Magnetic Resonance Imaging and Brain Informatics, Capital Medical University, Beijing, China; ^3^ Department of Obstetrics and Gynecology, Xuanwu Hospital, Capital Medical University, Beijing, China

**Keywords:** Lynch syndrome, 18F-FDG, PET/MR, endometrial carcinoma, colon carcinoma

## Abstract

**Background:**

Lynch syndrome (LS) is associated with the early onset of carcinoma and the development of numerous types of carcinoma, particularly endometrial and colon carcinomas. LS-associated endometrial carcinoma (EC) has been widely noted by gynecologists. However, there is still a lack of a non-invasive and reliable tool for early screening for LS in patients with EC. There are a few reports of PET and MR images revealing EC associated with LS.

**Case presentation:**

A 63-year-old female patient presented with postmenopausal intermittent vaginal bleeding. Transvaginal ultrasonography showed a small amount of bleeding in the uterine cavity and no thickening of the endometrium. The levels of relevant tumor markers were all within normal ranges. The endometrial cytology examination hint to possible endometrial adenocarcinoma. The hybrid 18F-fluorodeoxyglucose (18F-FDG) positron emission tomography/magnetic resonance (PET/MR) images showed a polypoid mass in the lower uterine segment and unexpectedly found a mass in the descending colon. A colonoscopy confirmed that there was a colon adenocarcinoma in the same place as the PET/MR images. Thus, LS was suspected even though this patient did not match the clinical diagnostic criteria. The gene analysis of both tumors was performed to identify microsatellite instability (MSI) for the diagnosis of Lynch syndrome. Postoperative adjuvant therapy and follow-up protocol customized for patients with Lynch syndrome.

**Conclusion:**

This case highlights that hybrid 18F-FDG PET/MR imaging could play a key role in the screening for Lynch syndrome in EC patients.

## Introduction

Lynch syndrome (LS) is an autosomal dominant carcinoma predisposition syndrome resulting from germline mutations of the DNA mismatch repair (MMR) genes, including MLH1 (mutL homolog 1), MSH2 (mutS homolog 2), MSH6 (mutS homolog 6), and PMS2 (postmeiotic segregation increased 2) (1, 2). It is related to the early onset of carcinoma and the development of many carcinoma types, particularly colon and endometrial carcinomas. Previous studies reported that 2%–5% of all endometrial carcinoma cases and 2%–3% of all colorectal carcinoma cases were associated with Lynch syndrome (3, 4). And LS-associated endometrial carcinoma (EC) has been widely noted by gynecologists, as females with LS have a 40%–60% risk of developing EC (5). Moreover, EC can act as a ‘sentinel’ carcinoma, leading to a 25% probability of developing a second carcinoma in the next ten years in patients with Lynch-associated EC (5). Early germline screening for Lynch syndrome in patients with EC or other high-risk populations could reduce the incidence of colorectal carcinoma by 5% to 29% and the mortality of EC by 7% to 42% (6). Therefore, early screening based on family history and pathologic features to identify Lynch syndrome has been encouraged by clinical experts to facilitate earlier treatment (6, 7). However, there is still a lack of a non-invasive, reliable, and inexpensive tool for early screening for Lynch syndrome in patients with EC.

This case shows that 18F-fluorodeoxyglucose (18F-FDG) positron emission tomography/magnetic resonance (PET/MR) examination could help a patient with EC screen for Lynch syndrome and modify their treatment plan.

## Case presentation

A 63-year-old female patient presented with postmenopausal intermittent vaginal bleeding for 2 months. She denied other urinary, rectal, and gynecological symptoms such as abnormal vaginal discharge, abnormal defecation, and abdominal pain. Transvaginal ultrasonography showed a small amount of hemorrhage in the uterine cavity, endometrial thickness of 0.68 cm, and no abnormal echo in the bilateral adnexal area. The levels of relevant tumor markers were all within normal ranges as follows: CA125:15.4 U/ml (reference value:<35 U/ml), CEA:2.21 ng/ml (reference value:<5 ng/ml) and CA19-9:13.6 U/ml (reference value:<37 U/ml). The endometrial cytology examination hint to possible endometrial adenocarcinoma. No other neoplasms, hereditary diseases, or related family histories were self-reported. The multimodal PET/MR examination was recommended to provide further information about the depth of myometrial invasion and the assessment of metastases in endometrial carcinoma.

**Figure 1:**
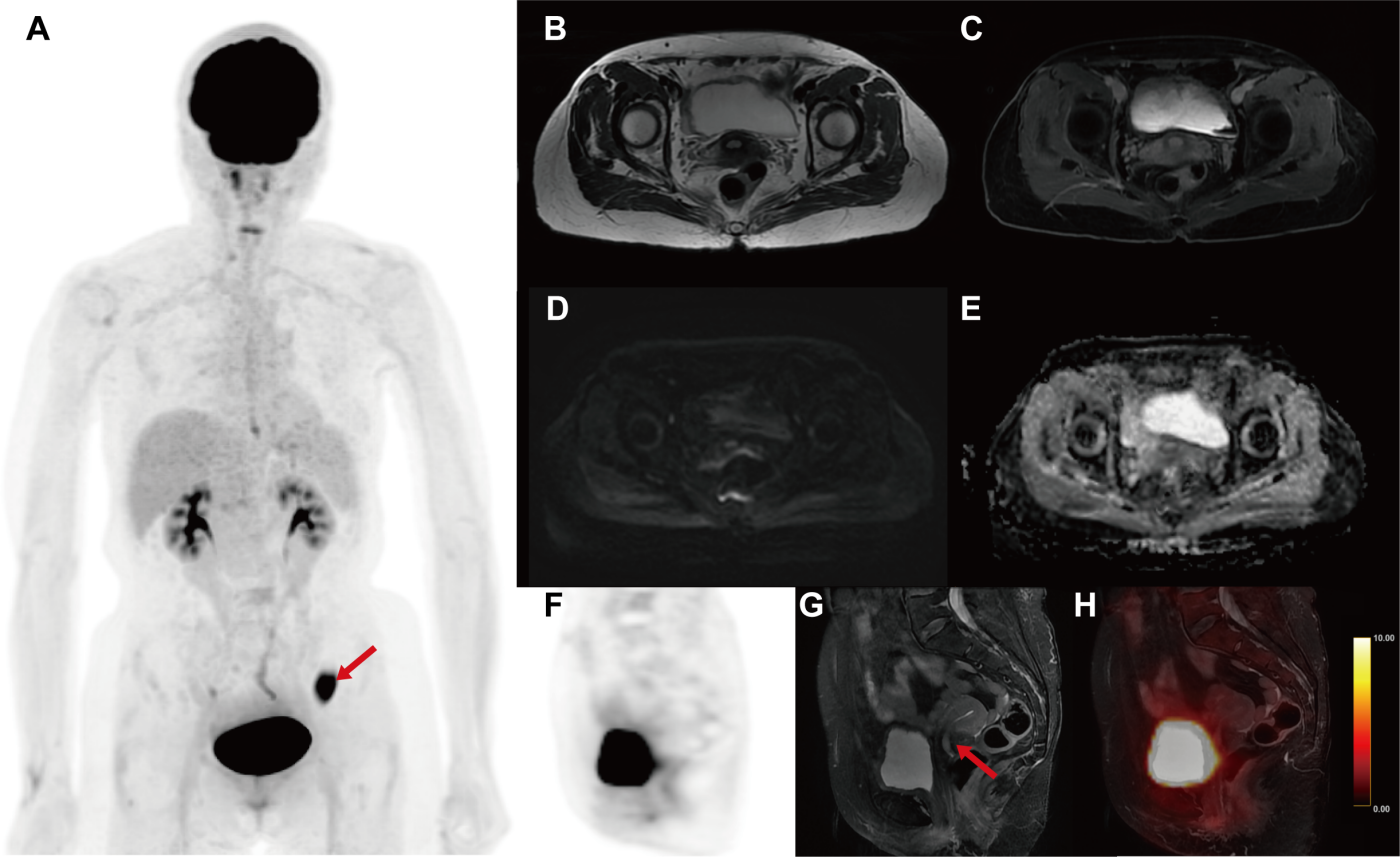
The multimodal 18F-FDG PET/MR images for evaluating the uterine. **(A)** MIP image of whole-body PET. **(B)** Axial T2WI. **(C)** Axial T1WI+C. **(D)** Axial DWI. **(E)** Axial apparent diffusion image (ADC) map. **(F)** Sagittal PET image. **(G)** Sagittal fs T2WI. **(H)** Sagittal PET/T2WI fusion image. On the pelvic sagittal MRI, there was a polypoid mass in the lower segment of the uterine (red arrow). The polypoid lesion showed a slightly high intensity signal on T1WI and fs T2WI, but no restricted diffusion signal in DWI and ADC. A mild enhancement of the lesion was observed on T1WI+C. The PET and PET/MR fusion images showed slight hypermetabolism in the lesion (SUV_max_ = 5.7g/ml). Unexpectedly, the MIP image of whole-body PET revealed an abnormal hypermetabolism in the left lower abdomen (red arrow, SUV_max_ = 17.68 g/ml).

The multimodal PET/MR imaging including the whole-body ^18^F-fluorodeoxyglucose (18F-FDG) PET and the multimodal MR images were performed with the hybrid PET/MR scanner (SIGNA TOF-PET/MR, GE Healthcare). The patient signed an informed consent form for the PET/MR examination and the publication of relevant images. On the pelvic sagittal MRI, there was no significant thickened endometrium and no high FDG uptake in the uterine corpus, but only a polypoid mass stretching from the lower segment of the uterine endometrium to the cervical canal (**Figures 1B, G**). The polypoid lesion showed a slightly high intensity signal on non-contrast T1-weighted images (T1WI) and fat-suppressed T2-weighted images (fs T2WI), but no restricted diffusion signal in diffusion-weighted images (DWI) (**Figure 1**). Following gadolinium administration, a mild enhancement of the lesion was observed on contrast enhanced T1WI (T1WI+C) (**Figure 1C**). The PET and PET/MR fusion images showed slight hypermetabolism in the lesion (**Figures 1F, H**). Based on the pelvic PET/MR multimodal images and cytology examination, the polypoid mass in the cervical canal was suspected to be endometrial carcinoma.

**Figure 2:**
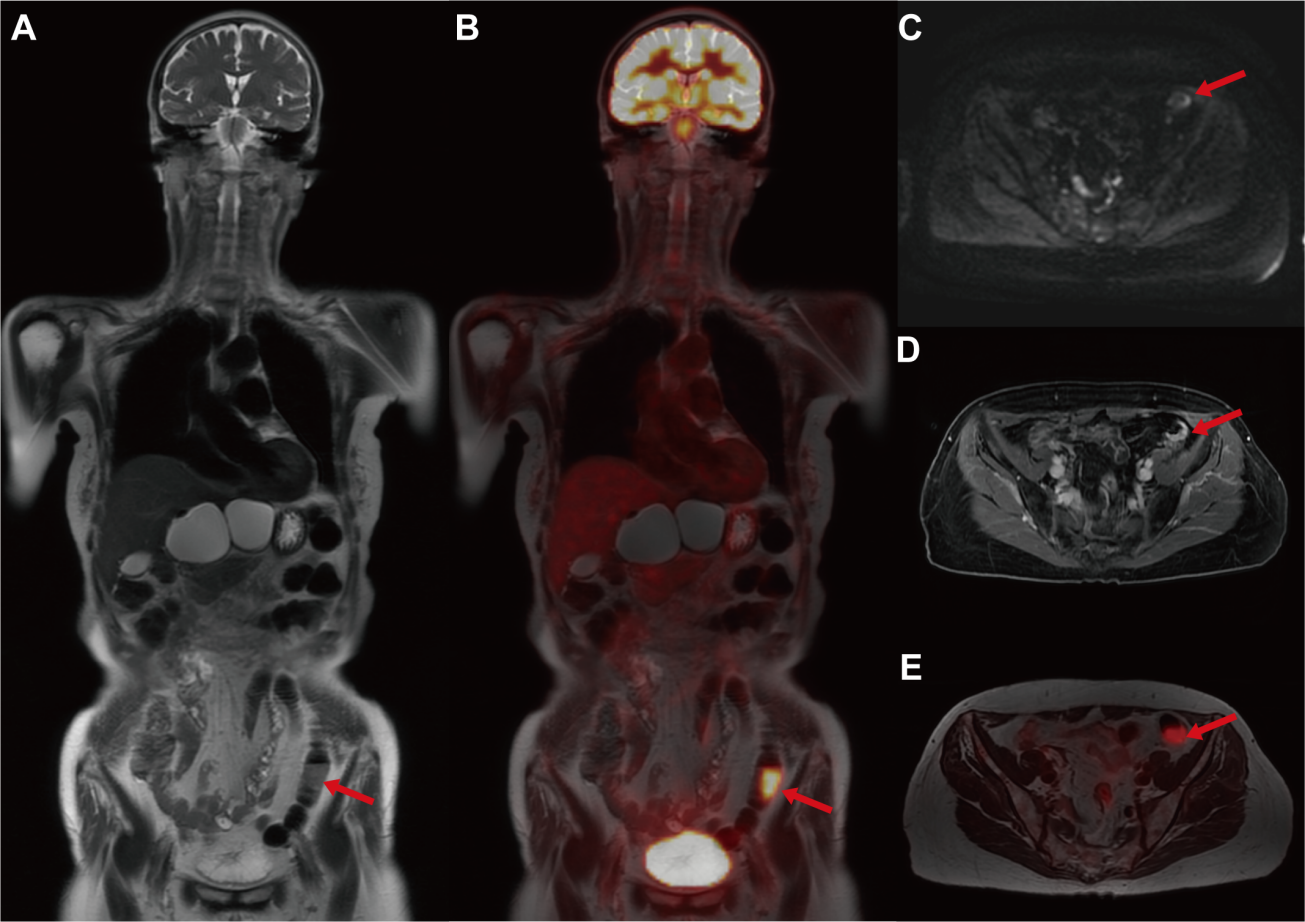
The multimodal 18F-FDG PET/MR images for evaluating the descending colon mass. **(A)** Coronal whole-body T2WI. **(B)** Coronal PET/T2WI fusion image. **(C)** Axial DWI. **(D)** Axial T1WI+C. **(E)** Axial PET PET/T2WI fusion image. The coronal whole-body T2WI and PET/MR fusion images showed that the abnormal hypermetabolism on PET images was a mass in the intestinal wall of the descending colon (red arrow). The thickened descending colon wall that showed markedly high signal intensity on either DWI (ADC_mean_ = 0.75*10^-3 mm2/s) or T1WI+C and hypermetabolism on axial PET/MR fusion images was observed (red arrow).

Unexpectedly, the maximum intensity projection (MIP) images of whole-body PET revealed an abnormal hypermetabolism in the left lower abdomen (SUV_max_ = 17.68 g/ml) (**Figure 1A**). Thus, the coronal whole-body PET/MR images were reconstructed to evaluate the abnormal hypermetabolism. The coronal whole-body T2WI and PET/MR fusion images showed that the abnormal hypermetabolism on PET images was a mass in the intestinal wall of the descending colon (**Figures 2A, B**). The thickened descending colon wall that showed markedly high signal intensity on either DWI (ADC_mean_ = 0.75*10^-3 mm2/s) (**Figure 2C**) or T1WI+C (**Figure 2D**) and hypermetabolism on axial PET/MR fusion images (**Figure 2E**) was observed.

**Figure 3:**
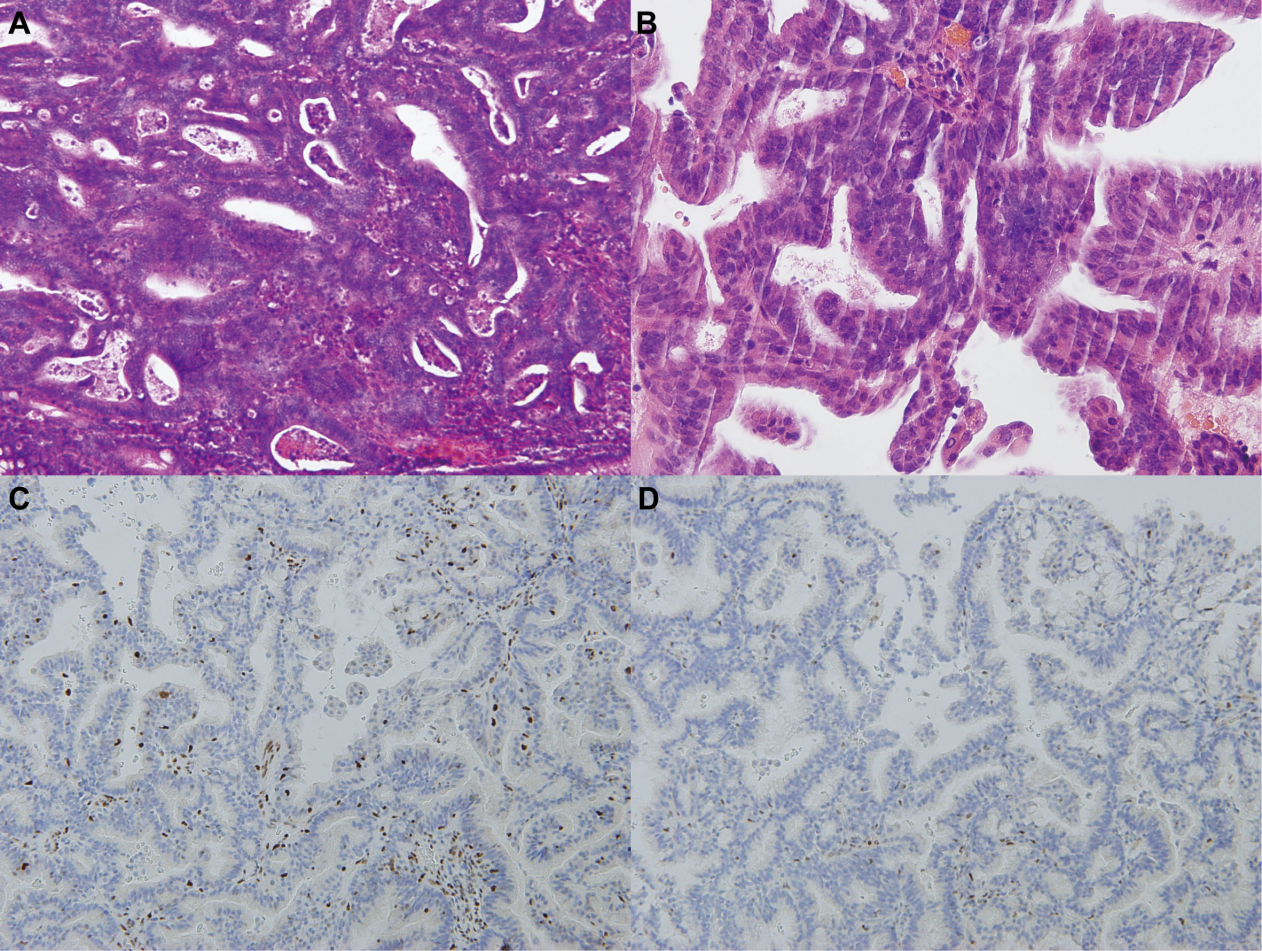
Histopathological images of endometrial and colon carcinomas. **(A)** The HE staining of colonic mass tissue in the descended colon (the microscope magnifying×400). **(B)** The HE staining of polypoid tissue in the lower uterine segment (the microscope magnifying×400). **(C-D)** The IHC staining of polypoid tissue in the lower uterine segment (the microscope magnifying×400). MLH1 and PMS2 proteins were negative expression in the IHC staining.

**Figure 4:**
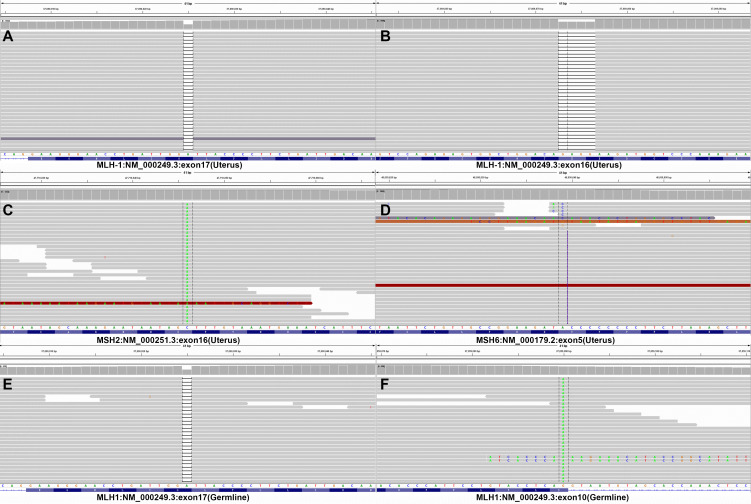
Gene analysis of endometrial and colon carcinomas. **(A-D)** The outcomes of DNA mismatch repair gene analysis in endometrial adenocarcinoma tissue. **(E-F)** The outcomes of DNA mismatch repair gene analysis in colon adenocarcinoma tissue. Endometrial adenocarcinoma tissue genetic testing revealed deletion mutations of exons 16 and 17, replacement mutations of exon 16 in MSH2, and duplication mutation of exon 5 in MSH6. Exon 10 and 17 mutations in MLH1 were also discovered during genetic testing of the colon adenocarcinoma sample.

Subsequent colonoscopy examination confirmed the presence of a descending colonic tumor on the corresponding location of PET/MR images. The patient underwent simultaneous laparoscopic radical hysterectomy and descending colectomy. Macroscopic analysis of the resected uterus confirmed that the polypoid mass stretching from the lower segment of the uterine endometrium to cervical canal. And the endometrial carcinoma (G2) (**Figure 3B**) categorized as superficial myometrial invasion and the poor-differentiated tubular adenocarcinoma (**Figure 3A**) invading the muscularis propria without serosa invasion were demonstrated by histologic analysis. Lynch syndrome was considered because the patient had both primary endometrial carcinoma and descending colon carcinoma, even though she had no relevant family history. Immunohistochemistry (IHC) analysis indicated that MLH1 and PMS2 proteins were negative expression in the resected tumor tissues derived from uterus (**Figures 3C, D**). Molecular analysis identified no KRAS and BRAF mutations of primary colon tumor. Moreover, the gene analysis of both tumors was performed to identify microsatellite instability (MSI) for the diagnosis of Lynch syndrome. The results of endometrial carcinoma gene analysis included deletion mutations in exon 15 and exon 17 of MLH1 (**Figures 4A, B**), replacement mutation in exon 16 of MSH-2 (**Figure 4C**) and duplication mutation in exon 5 of MSH-6 (**Figure 4D**). And deletion mutation and replacement mutation of MLH-1 derived from the colon tumor were also confirmed (**Figures 4E, F**). Therefore, post-operative adjuvant therapy and follow-up would be modified according to Lynch syndrome, and relevant genetic testing had been recommended for high-risk relatives.

## Discussion

Women with Lynch syndrome have a higher risk of developing EC than colon carcinoma, thus gynecological oncologists have recognized the importance of screening for Lynch syndrome in patients with EC (8). Young age of onset, more than one primary carcinoma in a person, and multiple family members with the same or related types of carcinoma are general hallmarks of Lynch syndrome as a hereditary carcinoma susceptibility syndrome (8). The current international clinical criteria and screening protocols for diagnosing Lynch syndrome mainly include the Amsterdam II criteria and revised Bethesda criteria (9, 10). These clinical diagnostic criteria for Lynch syndrome are based primarily on a history of relevant carcinoma in high-risk relatives and an age of onset of less than 50 years. However, the patient reported in this case did not fulfil the clinical diagnostic criteria and have any clinical signs associated with colon carcinoma, so we did not initially suspect Lynch syndrome. Hampel et al. also found that 60%–70% of endometrial carcinoma patients related to Lynch syndrome did not meet any clinical criteria by molecular analysis of 543 patients with EC (11). Most likely because these criteria focus on the diagnosis of Lynch syndrome in patients with primary colon carcinoma.

Many studies have proven the value of hybrid 18F-FDG PET/MR multimodal imaging for the diagnosis, Preoperative precise staging, and outcome evaluation of gynecologic malignancies (12–14). Whereas CT, MR, and PET are separate modalities, the integration of PET with MR allows anatomic and metabolic characteristics to be assessed simultaneously for local invasion and metastasis of endometrial carcinoma. In this case, a multimodal PET/MR examination was designed to assist in the assessment of local invasion and distant metastasis of EC. More notably, we also identified a localized intestinal wall thickening with increased metabolism in the descending colon by PET/MR multimodal images. Furthermore, a colonoscopic biopsy revealed that the mass in the descending colon was a poorly differentiated adenocarcinoma. On the other hand, EC was found in the lower uterine segment in this case, which is a common location for EC associated with Lynch syndrome. Westin et al. discovered the prevalence of Lynch syndrome in patients with lower uterine segment EC was much greater than that of the general EC patients or in endometrial carcinoma patients younger than age 50 years (15). It was revealed that 29% of women with lower uterine segment EC concurrently had Lynch syndrome. In contrast to the more typical EC found at the uterine body and fundus, EC obtained from the lower uterine segment is quite uncommon, accounting for only 3-8% of all EC (15). As a result, some case reports suggested that EC originating from lower uterine segment could indicate the presence of Lynch syndrome and could be used as evidence to screen for Lynch syndrome (16).

Due to the lack of criteria for Lynch syndrome screening in EC patients, the question of how and when to screen them has become urgent. Several case reports have shown that PET and MR imaging are beneficial for diagnosing Lynch syndrome and finding additional Lynch syndrome-related primary carcinomas in patients with EC (16, 17). In addition, a recent study demonstrated that the radiomics parameter derived from PET imaging could identify Lynch syndrome in patients with EC (AUC = 0.893) (18). And for EC patients with suspected concomitant colon cancer, PET/MR imaging might provide accurate local and distant staging both at the time of initial diagnosis and during follow-up compared to PET/CT images (19). Yu et al. found that the accuracy of PET/MRI was higher than that of PET/CT for early-stage EC diagnosis (86.0% vs. 77.2%) (20). Therefore, in this case, the diagnosis of Lynch syndrome was supported by the multimodal PET/MR imagings to precisely locate the endometrial carcinoma while also detecting the existence of a descending colon tumor. Mismatch repair gene mutations were ultimately validated in endometrial carcinoma samples by genetic testing in this case. Previous research suggested that females who had the MSH6 mutation had a higher risk of developing EC, whereas MLH1 and MSH2 were linked to an increased risk of colorectal carcinoma (21). The patient of this case with concurrent mutations in MLH1, MSH2, and MSH6 was consistent with having both EC and colon carcinoma, as determined by genetic analysis.

In this case, an unanticipated finding of colon carcinoma on 18F-FDG PET/MR examination led to the suspicion of Lynch syndrome. The information assisted the gynecologic oncologist in modifying the patient’s treatment plan. PET/MR multimodal imaging may be included as a screening tool in future clinical guidelines in addition to genetic testing.

## Conclusion

This case presented the incidental detection of descending colon carcinoma suggestive of Lynch syndrome by multimodal PET/MR images in a patient with EC. The hybrid PET/MR imaging that combines high-resolution structural imaging with metabolic imaging could play a key role in the screening for Lynch syndrome in EC patients.

## Ethics statement

Written informed consent was obtained from the individual(s) for the publication of any potentially identifiable images or data included in this article.

## Author contributions

Acquisition of data: XL, XZ, JM and BC. Manuscript writing: XL. Critical review of the manuscript: JL and SW. All authors contributed to the article and approved the submitted version.
